# Socio-environmental consideration of phosphorus flows in the urban sanitation chain of contrasting cities

**DOI:** 10.1007/s10113-017-1257-7

**Published:** 2017-12-19

**Authors:** Geneviève S. Metson, Steve M. Powers, Rebecca L. Hale, Jesse S. Sayles, Gunilla Öberg, Graham K. MacDonald, Yusuke Kuwayama, Nathaniel P. Springer, Anthony J. Weatherley, Kelly L. Hondula, Kristal Jones, Rubel B. Chowdhury, Arthur H. W. Beusen, Alexander F. Bouwman

**Affiliations:** 1 Department of Physics, Chemistry, and Biology (IFM), Linköping University, 581 83 Linköping, Sweden; 2 National Research Council, National Academies of Science, USA and School of the Environment, Washington State University, Pullman, WA USA; 3 Washington State University, Pullman, WA USA; 4 Department of Biological Sciences, Idaho State University, Pocatello, ID USA; 5 Department of Geography, McGill University, Montreal, QC Canada; 6 Institute for Resources, Environment, and Sustainability, The University of British Columbia, Vancouver, BC Canada; 7 Resources for the Future, Washington, DC USA; 8 Institute on the Environment, University of Minnesota, St. Paul, MN USA; 9 Faculty of Veterinary and Agricultural Sciences, The University of Melbourne, Melbourne, VIC Australia; 10 National Socio-Environmental Synthesis Center, University of Maryland, College Park, MD USA; 11 Department of Earth Sciences, Geochemistry, Faculty of Geosciences, Utrecht University, Utrecht, The Netherlands; 12 PBL Netherlands Environmental Assessment Agency, The Hague, The Netherlands

**Keywords:** Phosphorus, Sanitation, Sustainability, Socio-environmental factors, Urban

## Abstract

Understanding how cities can transform organic waste into a valuable resource is critical to urban sustainability. The capture and recycling of phosphorus (P), and other essential nutrients, from human excreta is particularly important as an alternative organic fertilizer source for agriculture. However, the complex set of socio-environmental factors influencing urban human excreta management is not yet sufficiently integrated into sustainable P research. Here, we synthesize information about the pathways P can take through urban sanitation systems along with barriers and facilitators to P recycling across cities. We examine five case study cities by using a sanitation chains approach: Accra, Ghana; Buenos Aires, Argentina; Beijing, China; Baltimore, USA; and London, England. Our cross-city comparison shows that London and Baltimore recycle a larger percentage of P from human excreta back to agricultural lands than other cities, and that there is a large diversity in socio-environmental factors that affect the patterns of recycling observed across cities. Our research highlights conditions that may be “necessary but not sufficient” for P recycling, including access to capital resources. Path dependencies of large sanitation infrastructure investments in the Global North contrast with rapidly urbanizing cities in the Global South, which present opportunities for alternative sanitation development pathways. Understanding such city-specific social and environmental barriers to P recycling options could help address multiple interacting societal objectives related to sanitation and provide options for satisfying global agricultural nutrient demand.

**Electronic supplementary material:**

The online version of this article (10.1007/s10113-017-1257-7) contains supplementary material, which is available to authorized users.

## Introduction

Urban populations consume increasing shares of food globally and produce large quantities of waste. With more than half of the global population now living in cities, there is an opportunity to couple sustainable food production and waste management systems in and around urban areas meeting multiple U.N. Sustainable Development Goals (SDGs) at once (e.g., sanitation (6) and sustainable cities (11) with food security (2)). Phosphorus (P) is a critical element at the nexus of agricultural production and waste management, but it is currently unsustainably managed in most food and waste systems (Chowdhury and Chakraborty 2016; Cordell and White 2014; Elser and Bennett 2011). Cities can play a key role in achieving global P sustainability, in part by recycling P from urban waste back to food production systems—without compromising human health through contact with pathogens in waste (Esrey et al. 2001). Conceptualizing and implementing P recycling approaches does, however, require that the variability among cities be taken into account as the magnitude, form, and locations of P flows vary widely across cities.

Organic waste management is not a new challenge for cities. Historically, some civilizations recycled their urban organic wastes, and the nutrients they contained, back to agricultural lands (Ashley et al. 2011). Today however, there is limited recycling in most urban settings; waste is disposed of by discharge to waterways, storage in landfills, or incineration, all of which have direct and indirect environmental impacts related to P (Chowdhury and Chakraborty 2016; Chowdhury et al. 2014). Several factors contribute to this situation including the separation of rural and urban living, economic viability, human health issues, and cultural attitudes and values such as acceptance of excreta recycling strategies (Garnier et al. 2015; Ma et al. 2014).

Human excreta is a significant component of urban organic waste streams and contributes to freshwater and coastal eutrophication (Carpenter 2008; Diaz 2001), especially where human waste goes untreated (Harrison et al. 2010). Globally, P discharge from urban areas to waterways has increased more than 4.5-fold since 1900, in line with population increase, while the quantity of P recycled back to agricultural lands is estimated to have increased a mere 14% (0.07 to 0.08 Tg P year^−1^, Morée et al. 2013; Van Drecht et al. 2009). Recent estimates claim that 22% of the global P fertilizer demand can be met by recycling P from urban human excreta (urine and feces) to agricultural lands (Mihelcic et al. 2011). Harnessing this potential could also divert excreta from waterways and contribute to food security. Understanding the social and environmental factors that facilitate and constrain P recycling, especially the complex factors specifically linked to human waste (Jewitt 2011), is therefore critical for future sustainability.

Despite the great potential for urban P recycling, the magnitudes and destinations of P flows at the city level and their heterogeneity across cities remain poorly understood. Current estimates of P in urban human excreta flows largely rely on regional or national assumptions about food consumption patterns and waste treatment. For example, Van Drecht et al. (2009) used income as a proxy for P consumption when data were not available nationally and regional World Health Organization data to model connections to sewerage systems and treatment. Building on this, Moree et al. (2013) created models of the fate of urban wastes based on regional assumptions about changes in waste management practices through time. To our knowledge, these are the most comprehensive global studies on urban nutrient dynamics over time, which elucidate important global spatial patterns of P from urban wastes. It has simply not been possible to carry out comparative global studies that quantify urban P recycling at a finer resolution than the national scale due to limited high resolution and global coverage data on urban P flows. Existing global studies also use social and built infrastructure factors to calculate estimates of P flows, which highlights the general understanding that these factors are important but prevents using the factors to test their importance because of circular logic. Detailed case studies are needed to create independent estimates of P recycling that can be used to evaluate how local socio-economic and biophysical factors influence recycling.

Urban P case studies must consider a city’s diverse cultural, environmental, and infrastructural legacies, as well as growth and transformational plans (Childers et al. 2014). Metson et al. (2015) identified eight socio-environmental dimensions that need to be considered when evaluating alongside urban P flows. These include not only direct factors, such as infrastructure and financial resources, but also more indirect drivers that should be considered as part of the network of factors that creates the socio-environmental context within which P flows exist. For example, the geographical distance to coastal waters or large rivers could be an important indirect factor to consider. Distance from water not only affects a city’s capacity to discharge human excreta to such bodies of water but also may influence water availability for irrigation in the region, indirectly motivating the recycling of nutrients present in wastewater (e.g., Metson et al. 2012a). To harness global urban P recycling potential, it is necessary to account for city-specific socio-environmental factors that affect P flows through urban sanitation systems. We argue that identification of these factors could allow for better alignment between P sustainability and existing urban priorities and to better determine how cities can learn from each other’s successes and failures.

A comparative social-ecological analysis of urban P flows is methodologically challenging however. Quantifying urban P flows and qualifying socio-environmental context for even one city are difficult as city-level data sources remain relatively fragmented and unstandardized. Comparisons between cites only adds to this complexity and represents an obstacle to urban P research. Available and relevant data sources vary among cities, and at times, the reasons for differing data sources overlap with the socio-environmental context that needs to be investigated. For example, differences in funding levels for centralized sanitation can result in different levels of publicly available reporting. In addition, discrepancies in how urban boundaries are defined can further challenge our ability to generate comparable datasets (noting however, that Chowdhury et al. (2014) have performed a systematic qualitative review of P flow studies that provides a basis for an indirect comparison). Even if challenging, comparisons across cities are essential to develop, test, and critique potentially generalizable urban sustainability and urban ecology theories. A synthesis approach can help overcome these challenges by pulling together different data sources and information across cities in different contexts (Palmer et al. 2016). Such a synthesis can help identify when cities face similar opportunities and challenges.

We ask the question: How do human excreta P recycling flows, sanitation chains, and associated socio-environmental factors differ across cities?

Here, in order to compare P recycling and socio-environmental factors which create constraints and opportunities for such recycling across cities, we apply a flexible, yet systematic and consistent, framework to five case study cities. More specifically, we adapted the sanitation service chain (e.g., World Bank, 2014) to P flow analysis in order to estimate the proportion of P from human excreta currently recycled back to agricultural lands in each city. We then combined these numbers with Metson et al.’s (2015) framework on socio-environmental factors to identify drivers and barriers to recycling in each city. We expected that cities would have different constraints and opportunities shaping the levels of recycling observed (e.g., as water availability or cultural acceptance mentioned above). We then identified where there were similarities and differences between cities as a step towards a better understanding of P management options that may be transferrable among cities, as well as highlight a fuller range of opportunities and challenges facing urban P recycling as there are no panaceas (Ostrom 2007).

## Case study selection, data collection, and framework adaptation

We used a purposive data collection strategy to identify five cities (Teddlie and Yu 2007) and compiled qualitative and quantitative information for each selected city: Accra, Ghana; Buenos Aires, Argentina; Beijing, China; Baltimore, USA; and London, England. We selected cities that represent a spectrum of economic and infrastructure development, different political contexts, and different environmental settings (notably aridity) on different continents. Further, information on sanitation management and/or P flow was available from the primary and gray literature for these case studies. The sanitation context of these cities varies widely, for instance, from Buenos Aires, where many underserviced poor areas utilize on-site solutions, to London, where the entire metropolitan area is connected to a central sewer system.

To develop the case studies (Yin 2013), we collected information on human excreta flows and contextual information on socio-environmental factors influencing flows by reviewing and synthesizing diverse data sources, which varied by city. Sources included peer-reviewed academic papers, government reports, and gray literature from NGOs and utilities. News articles were used in several cases as well. Site-specific information was complemented by national averages when local data were not available. Because different literatures and policy documents use different terms to describe sanitation systems, we provide an overview of terminology in Box SI1.

**Fig. 1:**
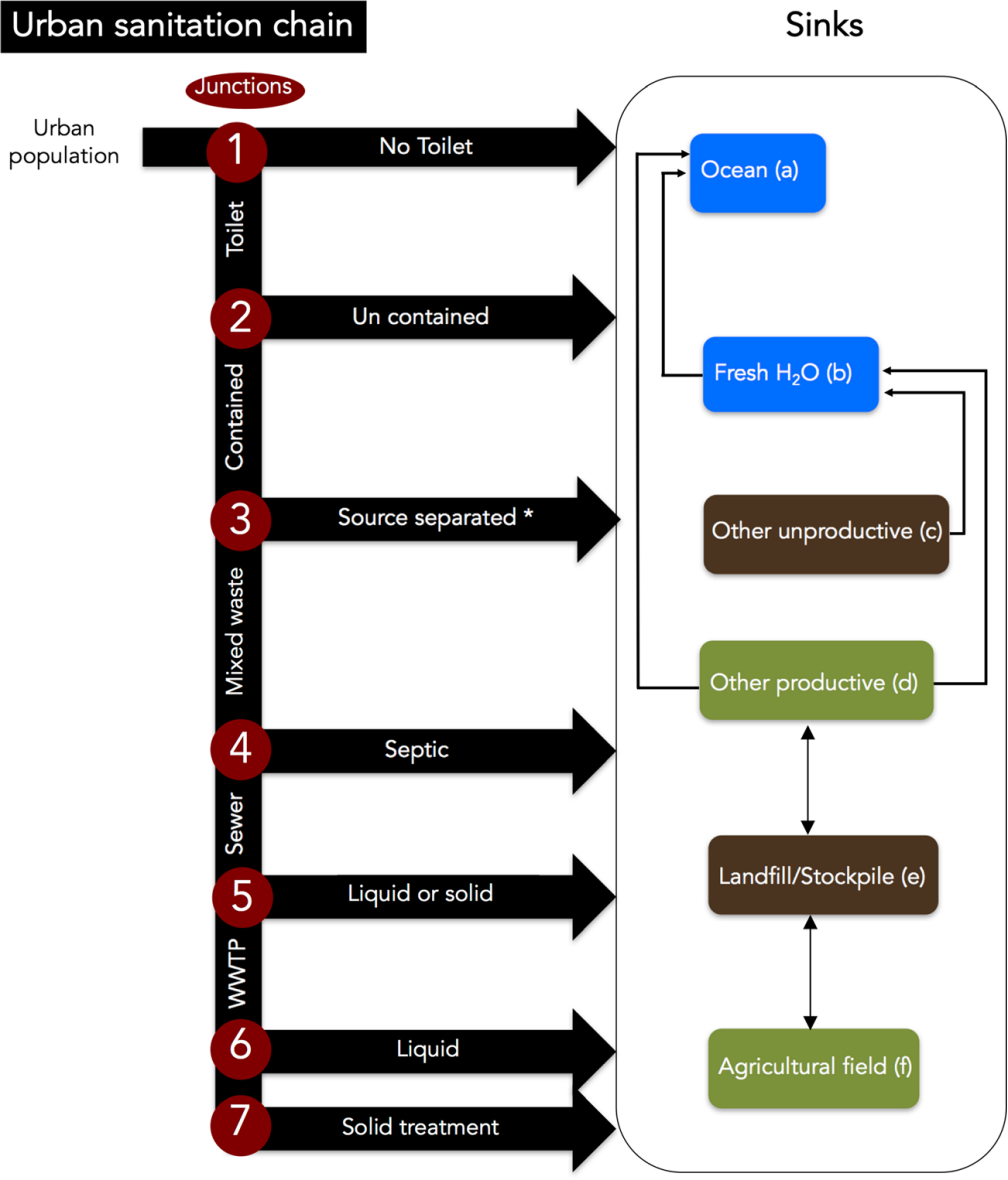
Conceptualization for tracking P through the urban sanitation chain. Each junction is numbered and each sink (i.e., receiving systems) lettered. This allows one to both quantitatively track P through the system as well as gain insight on the socio-environmental factors that affect each node and thus the fate of P through the urban system. At any of these junctions, P can exit the sanitation service chain creating seven paths towards one of six sinks: **a** the ocean, **b** fresh water bodies, **c** other unproductive uses besides landfill (e.g., uncultivated soil), **d** other productive uses besides agriculture (e.g., reforestation and land reclamation projects, or landscaping), **e** landfills or stockpiles, and **f** agricultural fields. Note that all arrows of sinks to fresh water are also potentially present for ocean but we simplified here as it is more likely that that sinks (**c**, **d**, **e**, and **f**) are located close to fresh or ground water than to the ocean globally.* *Junction 3* (i.e., source separated waste collection) have similar downstream junctions as 4, 5, 6, and 7 but we have simplified the P-sanitation service chain for ease of reading

Although data sources varied by city (see Table 1), we adapted the sanitation service chain framework, which is commonly used to analyze the flow of pathogens associated with urine and feces (World Bank, 2014), in order to organize data consistently and make sure the scope of our assessments where comparable. Figure 1 illustrates our application of the sanitation service chain to urban P flows. In our approach, the “P-chain” begins with the total urban population, which generates a flow of human excreta. We distinguish seven “junctions” where the flow of P in excreta is partitioned into different pathways. At any of these junctions, P can enter one of six sinks: (a) coastal waters or the ocean, (b) fresh water bodies, (c) other unproductive areas besides landfill (e.g., uncultivated soil), (d) other productive areas besides agriculture (e.g., reforestation and land reclamation projects, or landscaping), (e) landfills or stockpiles, and (f) agricultural fields. For example, sewage that is not treated at a treatment plant because of stormwater overflow at *Junction 5* constitutes “leakage” from the sanitation chain towards waterways. There also can be flows between the sinks. For example, P applied to agricultural fields (f) can leak into freshwaters (b) and in turn motivate regulations that influence other P flows, such as the apportioning of P at *Junction 6*.

We applied the sanitation chain heuristic to identify factors, from the eight categories identified by Metson et al. (2015), driving/influencing P flows in each city. Applying this heuristic assured that we investigated each junction and how drivers may affect downstream junctions. The approach was particularly useful in order to move beyond direct, often technological or infrastructural, drivers. For example, *Junction 1*, as well as P flows at other downstream junctions, would be affected by cultural norms regarding open defecation. Junctions 4 and 5 might be significantly affected by land use such as large non-residential tracts of land separating housing from the city center and existing sewer infrastructure and wastewater treatment plants.

**Fig. 2:**
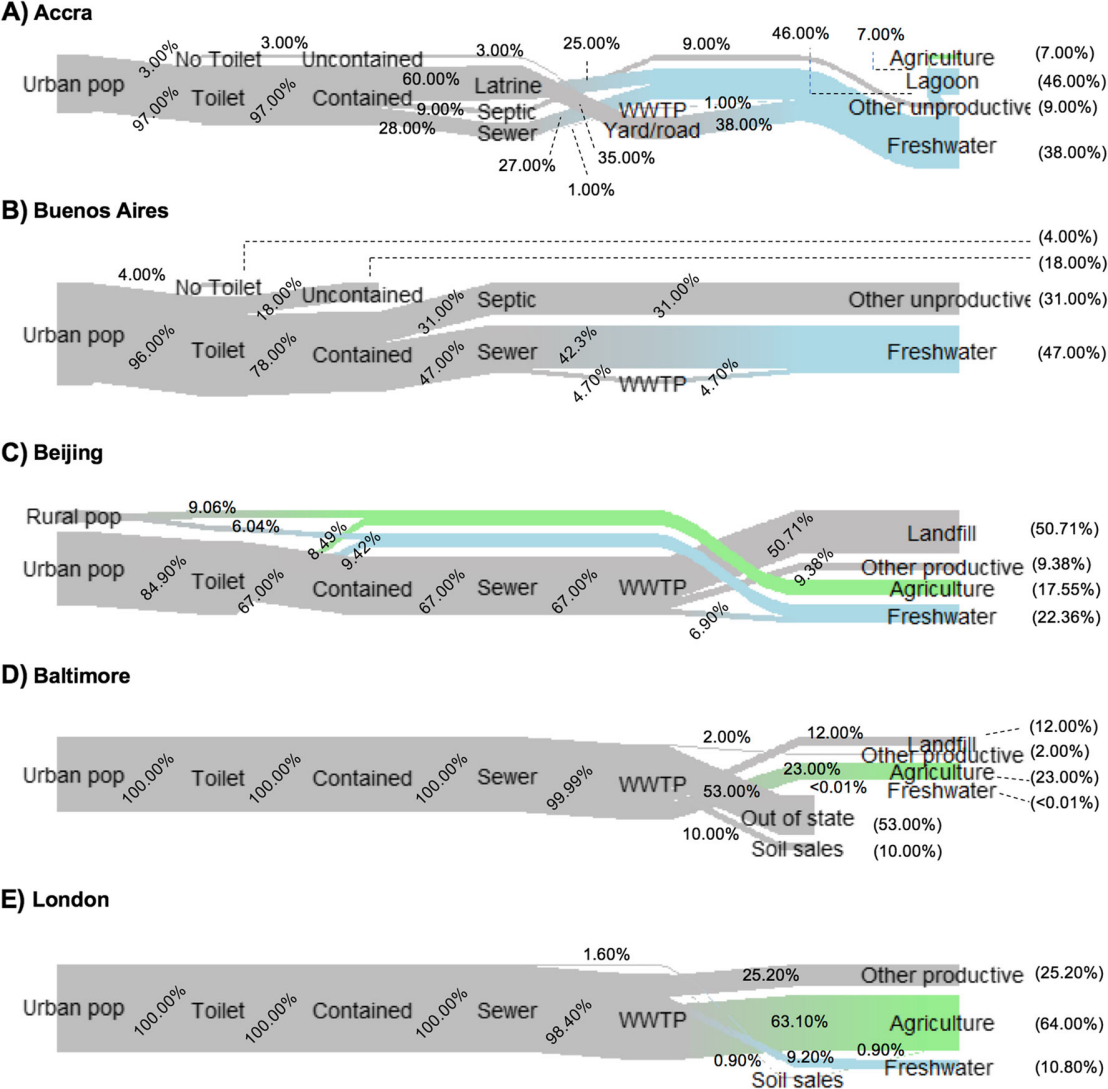
Flows of P from each city’s population through the sanitation system in **a** Accra, Ghana, **b** Buenos Aires, Argentina, **c** Beijing, China, **d** Baltimore, USA, **e** London, England based on the conceptualized P-sanitation service chain in Fig. 3. In order to standardize the results and account for differences in the types or amount of information available for each city, the flows are shown in proportion to the total population of each city. The transition from gray (urban source) to colors (sinks) indicates important “source to sink” paths, where blue indicates losses or disposal to water, while green indicates recycling to agricultural land

We first applied the P-sanitation service chain to each city. Our findings are presented as simplified P flow Sankey diagrams (Fig. 2, made with the riverplot package in R (Weiner 2015)), which depict the proportion of P in urban human excreta flowing through each of the seven junctions from the P-sanitation service chain (Fig. 1). We depict these flows as proportions, instead of quantifying site-specific flows, because not all cities had sufficient P-specific information to facilitate comparison across different size cities. We then reviewed these P flow diagrams through the lens of Metson et al. (2015)’s urban P framework to uncover which socio-environmental factors may have influenced the dominant P flows (magnitude and direction) at each of the seven junctions and six sinks of the P-chain. We use a narrative structure to convey the socio-environmental context based on the diverse quantitative and qualitative data available for each city.

## Results: synthesis of P flows and socio-environmental factors in five case study cities

The cases are presented by order of increasing connections to a centralized sewer system and increasing level of treatment at centralized treatment plant(s) (Fig. 2). Each narrative (Polkinghorne 1995) presents basic information on the city, briefly highlights the main path(s) through a Sankey diagram (Fig. 2), puts forth the most important socio-environmental factor(s), and identifies sanitation and P recycling opportunities and challenges. A more comprehensive explanation of the proportional flows and their socio-environmental drivers, as well as a land use and biophysical comparison among cities, is provided in the SI.

### Accra, Ghana

Accra is a rapidly growing coastal city of 4 million people with limited public or private capital (Boadi and Kuitunen 2005; GSS 2013; Nikiema et al. 2013; Nimoh et al. 2014).

Based on our information, the two dominant paths for excreta P in Accra are as follows:

- Latrines → yard/road → ocean
- Latrines → lagoon → ocean

Numerous priorities related to social and economic self-preservation take precedence over sanitation needs (Nimoh et al. 2014). The central sewer system is poorly developed, and most of the fecal sludge is handled on a day-to-day basis with practices such as door-to-door waste collection, disposal in communal collection points, or direct release into waterways (Boadi and Kuitunen 2005). Despite some negative perceptions about using human excreta as fertilizer (Diener et al. 2014), surface water containing untreated waste is currently being used for irrigation (Diener et al. 2014; Van Rooijen et al. 2010), likely driven by water scarcity (Owusu et al. 2012). Farmers in peri-urban Accra not only tend to view human excreta as a resource for agriculture but also express concerns for health risks, and it appears likely that these concerns limit wider P reuse (Nimoh et al. 2014, Shai-Osudoku district 400 farmer survey). The comparatively high cost of commercial fertilizers and the agronomic benefit of human excreta to farmers in Ghana have been found to motivate reuse in some contexts, for example with young farmers that own their own land (Cofie et al. 2010). In other areas of the country, where human excreta are considered a waste product, reuse is virtually unheard of (e.g., the city of Efutu (~ 160 km west of Accra near the coast), Mariwah and Drangert 2011).

Key challenges and needs in Accra are to improve sanitation for public health, but capital is scarce; the Ghanaian government has set ambitious targets for increasing access to sewer connections, especially for the Accra region, with less than 20% of the population having sewer access (ADF 2015, GSS 2013). Multiple projects with funding from international sources are underway but are not progressing as rapidly as hoped, serving only parts of the city as of 2015 (Quaye 2015). Still, progress is being made: a new wastewater treatment plant at Lavender Hill (a site where untreated waste was dumped for many years) became operational in 2017 (Government of Ghana 2017, Mbugua 2017). The use of untreated surface water containing human excreta for irrigation of food crops currently recycles P but may not be sustained owing to safety concerns for both farmers and consumers (Van Rooijen et al. 2010).

### Buenos Aires, Argentina

Buenos Aires is the largest city in Argentina at approximately three million people within the city and 13 million in the metropolitan area (Öberg et al. 2014).

Dominant paths for excreta P in Buenos Aires are as follows:

- Toilets → sewer → river (fresh H_2_O) → ocean
- Toilets → septic/pit-latrine → non-agricultural soils or river (fresh H_2_O) → ocean

The sanitation system has led to extreme pollution of the local rivers (50% of the flow in the Matanza-Riachuelo river is sewage). Major efforts have been made to improve the situation and the present plan is to see all households are connected to the central sewer system by 2020 (AySA 2010). The plan is to move sewage discharge from local rivers to Rio de la Plata (a river with an extremely high flow rate) as discharge to the local rivers is seen as highly problematic (Öberg et al. 2014). Progress is slow, however, and prior studies suggest that the ongoing lack of progress in expanding sewer connectivity and wastewater treatment is linked to institutional fragmentation and instability (Botton and De Gouvello 2008). It is not surprising that there is widespread doubt that full sewerage coverage will ever be achieved (del Carmen Morales et al. 2014; Merlinsky 2013; Öberg et al. 2014).

Local organizations appear to be lobbying for smaller treatment facilities and hybrid centralized and decentralized solutions to avoid leakage of human excreta (Öberg et al. 2014). However, there is no tradition of urban farming, and the soil in areas lacking a sewer connection may be polluted, perhaps necessitating container-based cultivation. There is no regulation against land application of recycled human excreta, but it is not a socially acceptable practice (Merlinsky 2013 and pers. comm.). If the city meets its strategic plan goal of connecting 95% of the population to the central sewer system (AySA 2010), this could lead to more challenges. Sludge storage is currently done in low-lying areas prone to inundation, and increasing storage at these locations would simply exacerbate the risk of nutrient losses during inundation. Increasing sewer connection rates may also not result in more productive P recycling. Intensive agricultural areas in the upstream part of Buenos Aires’s drainage basin already struggles with nutrient leakage, and thus increasing sewer connections to centrally collect waste and recycle it to peri-urban areas could exacerbate this issue (Öberg et al. 2014).

### Beijing, China

Beijing is the second most populated city in China, with nearly 22 million people living in the metropolitan area (UN Habitat, 2016).

Dominant paths for excreta P in the central city are as follows:

- Toilets → sewer → WWTP → landfill

This semi-arid region has experienced prolonged periods of drought coupled with major recent population growth (Sun et al. 2014). These pressures have prompted Beijing’s government to take an aggressive stance on wastewater reuse (Sun et al. 2014; Zhang et al. 2015), which affects wastewater infrastructure development and the potential for P recycling. Hosting the 2008 Olympic Games was another catalyst for the development of several new WWTPs; however, several of these are now operating beyond capacity due to continued population growth (Kuang 2012; Pernet-Coudrier et al. 2012). Many WWTPs do not meet environmental and health standards, despite aggressive local policies and aspirations for increasing wastewater reuse (Jia et al. 2011; Sun et al. 2014; Zhang et al. 2015). River water quality also falls below government standards (Zhang 2015). In addition, although central city residents are connected to central sewage, sanitation infrastructure in Beijing suburbs seems highly variable, with both the municipality and local newspapers highlighting recent efforts to increase access to toilets and reduce open defecation and latrine use (Beijing Municipal Government 2014; Hu 2015). While urbanization has replaced large amounts of agricultural land in Beijing over the last three decades (Kuang 2012), roughly 394,100 ha remains (Irie et al. 2014), where urban P can be recycled locally (including both urban and rural areas within municipal limits).

Strong state control is a distinctive theme in the case of Beijing, and this is likely to continue affecting P recycling issues in the future. Developing a “clean modern city” is a priority for the local government, which recently dedicated 1.8 billion Yuan ($27 million USD) as part of an urban cleanup initiative that includes sewage treatment and wastewater recycling (Beijing Municipal Government 2014). In contrast, rapid growth and ambiguous land rights and state responsibilities in peri-urban Beijing make P flows in these areas look more like those in Buenos Aires and Accra, where there is neither centralized infrastructure nor government accountability to improve waste management (Wu et al. 2013).

### Baltimore, USA

Baltimore, a city of 620,000 people (U.S Census Bureau 2016), is situated within the most urbanized region of the U.S., the Northeast Megalopolis, which includes New York City, Philadelphia, and Washington D.C.

The two dominant paths for excreta P are as follows:

- Toilet → contained sewer → WWTP → agricultural land
- Toilet → contained sewer → WWTP → exported out of state

The city is located on the Patapsco River arm of Chesapeake Bay and is a good representation of the situation facing older U.S. cities under both local and national pressure to improve their sewage management (Pelton et al. 2015). The current sewer system was constructed over 100 years ago and provides 100% coverage but has deteriorated with time and is increasingly costly to maintain (Boone 2003). Both local and national government regulations and laws are in place to discourage pollution and incentivize capture and reuse, stimulating technological solutions for recycling P back into productive uses (Coale et al. 2002). State level permits and bonding mediate sewage sludge allocation, while the U.S. Environmental Protection Agency and local initiatives pressure the rehabilitation of sewage infrastructure (EPA 2002, Healthy Harbor Initiative 2015). There is also pressure from citizens to address combined sewage overflows (CSOs) and sludge application issues in agricultural areas and disadvantaged communities, where complaints of smell, public health, recreation, and threats to biodiversity are still prevalent (Hare 2007; Pelton et al. 2015). There is a perception within Baltimore communities that pollution management in the past has adversely impacted the urban poor and could continue to do so in the future (Boone et al. 2009). As a consequence, future P innovations around Baltimore are likely to be viewed with major skepticism from the public, particularly in low-income communities that have been negatively impacted in the past, both directly through excess pollution and indirectly through associated impacts such as decreased real estate value and increased poverty and crime.

### London, England

Currently, the Greater London population stands around 8.6 million, although the city does sit within an even larger metropolitan area of a total of 14 million people (Office of National Statistics (ONS), 2016).

The dominant path for excreta P in London is:

- Toilet → sewer → WWTP → agricultural land

The current pathway of P from excreta through the city appears to be influenced by the long-standing sanitation infrastructure of the city and more modern environmental regulations. Development of sewage systems in London was largely a response to “The Great Stink,” where hot weather exacerbated the smell of untreated waste in 1858 (Halliday 2001), along with more concerning cholera outbreaks (Cicak and Tynan 2015). The dramatic reduction of cholera epidemics set a global example for urban wastewater innovation and implementation (George 2008; Johnson 2006), although it was not the first city to implement innovative human excreta and water management.

Because biosolids also contain nitrogen, the application of treated human excreta (biosolids) to land has been highly regulated both at the national (Department for Environment Food and Rural Affairs (DEFRA), 1990) and the EU level (e.g., Directive 86/278/EEC), through policies such as the Sewage Sludge Directive, especially in “nitrate vulnerable zones,” around London (Thames Water Utilities 2008; Worrall et al. 2009). Future challenges for P recycling in Greater London include ongoing population growth, soil P saturation, limited availability of receiving lands, and mixed public perceptions about recycling (Kelly et al. 2002). Most of the treatment facilities currently produce a digested biosolid product that is reapplied to fields (or in land reclamation). In addition, business partnerships are being forged with London utilities to produce more refined fertilizer P products from wastewater, such as struvite crystals (BBC News online 2013), and to reuse P containing ash from power generation at plants that will be marketable farther away from the London area.

### Case study summary

As expected, we find that generalized pathways of P through the urban sanitation chain vary widely across cities, which in turn influences P recycling rates. Recycling of P ranges from 0% in Buenos Aires to over 70% in London. Some variation can be attributed to infrastructure and capital. Baltimore and London have full toilet access, containment, treatment, and high rates of recycling, whereas Accra and Buenos Aires have comparatively less access to flush toilets, centralized collection, and less treatment. Beijing is a complex mixture, with nearly complete treatment in the central city but simultaneous growth of quasi-legal settlements with little infrastructure investment. Infrastructure availability directly affects the flow of P through each city and more infrastructure and containment at upstream junctions translates to more recycling of P downstream. However, it is necessary to move beyond these direct influences to understand the network of socio-environmental factors that indirectly shape our capacity for sustainable change.

There are substantial differences in P recycling rates and dominant pathways across cities that cannot be attributed to differences in economic and infrastructural development alone. For example, Accra and Buenos Aires have relatively low access to centralized sewer systems (15% in Accra and 55% in Buenos Aires) and water treatment plant capacity is severely limited in both cities. Both cities are challenged by rapidly growing populations, largely in informal settlements, which do not have access to adequate public or private sanitation services. There is no recycling of P in Buenos Aires, whereas in Accra, the dry climate and scarce water supplies have led to low rates of informal recycling, as urban farmers use water containing human excreta flowing through urban ditches to irrigate their crops. On the other hand, Buenos Aires has easy access to coastal waters via the Rio de la Plata. Ample water access, with sufficient flow, makes waste disposal easy, achieving desired health benefits of sewage disposal and potentially disincentivizing the higher costs of treatment and recycling. This comparison highlights that biophysical factors, which affect other urban priorities such as access to water for irrigation or safe disposal of waste, intermingle with capital and infrastructure drivers, and play an important role in understanding recycling rates.

## Discussion

### Socio-environmental factors constrain patterns of P recycling across cities

**Fig. 3:**
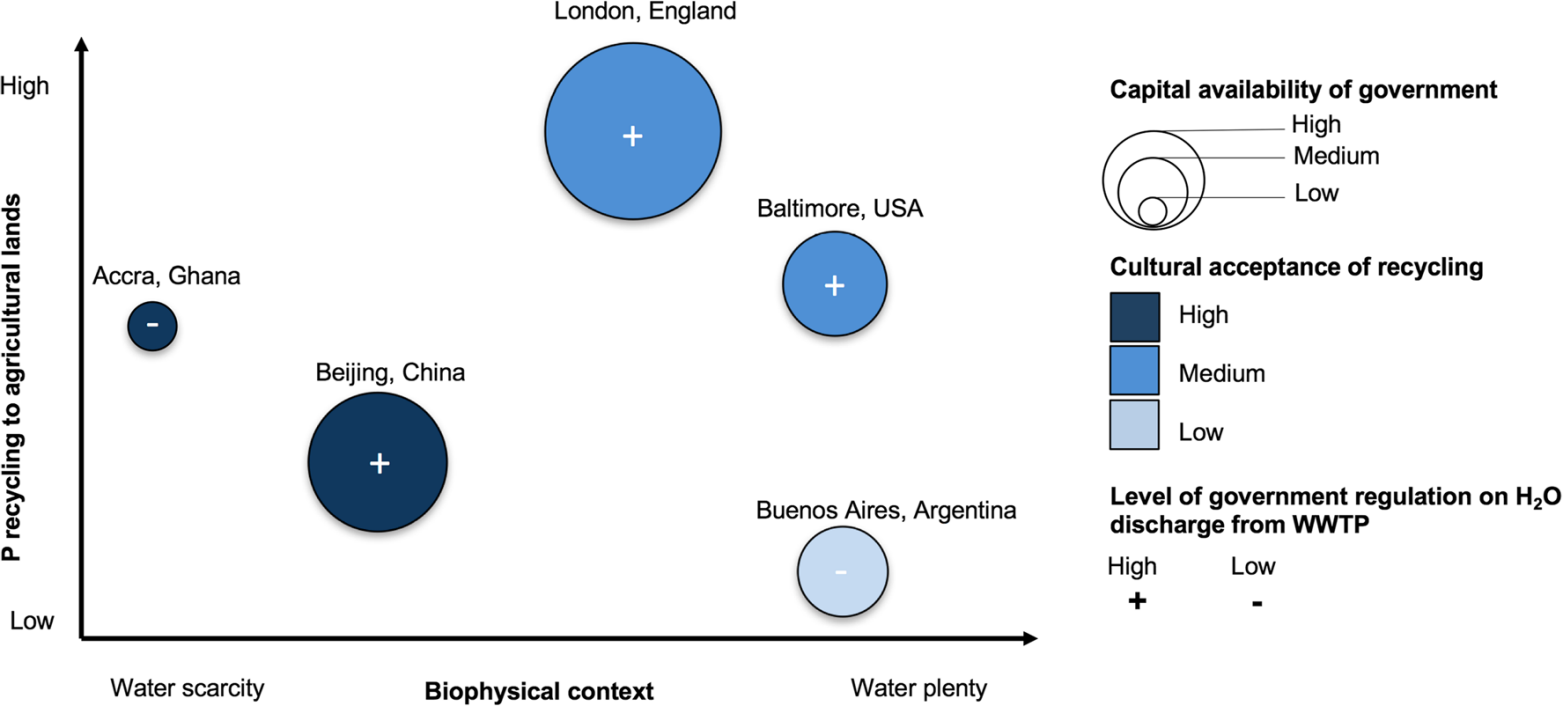
Illustrations of socio-environmental factors relevant to P recycling rates. Here we depict 1 water scarcity as a *biophysical situation* factor (*x* axis), 2 cultural acceptance of recycling as a *cultural norms and preferences* factor (light blue means less acceptance than dark blue), 3 monetary capital availability to government as a *market and capital availability* factor (smaller bubble means less capital than large bubble), 4 level of government regulation on H_2_O discharge from WWTP as a *government and regulation* factor as potentially interacting and affecting 5 P recycling (*y* axis). Italics here represent the categories of factors as defined by Metson et al. (2015). These four factors represent examples, are not the exhaustive list of factors affecting P recycling rates, and serve to demonstrate that no one factor is driving the observed pattern in recycling rates across the case study cities

Social and environmental factors that constrain or shape P pathways across our five study cities include infrastructure, capital, biophysical context, governance, and regulation. However, the relative importance of different socio-environmental factors affecting human excreta management across cities varies considerably. We therefore find that there are potentially necessary conditions to facilitate recycling but that no single socio-environmental factor seems sufficient to drive high recycling (Fig. 3).

High capital availability and regulation equate to higher recycling in London, but these factors do not translate across all cities (Fig. 3). Beijing, has invested relatively large amounts of capital in sanitation and heavy regulation, but the city has not achieved high rates of P recycling. Baltimore also has centralized infrastructure and strong environmental regulations but send much of its waste to landfill. EU environmental law strongly supports recycling of waste (while ensuring minimal health and recreation impacts on communities) as opposed to landfilling, which may explain this difference. London also has a larger proportion of nearby agricultural lands (SI Fig 1), where P can be recycled, so distance to recycling source may be a factor. Local soil P concentrations may also play a role. Both Baltimore and London have laws to limit P application where soil P is high to avoid losses from runoff and soil erosion.

The wider biophysical context, such as aridity, surely plays a role as well. Recycling in Accra seems to be motivated in part by water scarcity for urban and peri-urban farmers. This local recycling can be compared with another arid region, Phoenix, AZ, where P is indirectly recycled (although highly regulated and managed) through wastewater reuse because of water scarcity concerns (Metson et al. 2012a; Metson et al. 2012b). This observation also illustrates how P recycling cannot be divorced from other issues such as water management.

Among the case studies, Beijing stands as an outlier. For example, rapid top-down capital investment in centralized sewage has not resulted in equal sanitation benefits to all citizens or in high P recycling from treatment plants, and rural populations may still be recycling P back onto agricultural fields despite rapid urbanization (Ma et al. 2014). Additionally, the region’s semi-arid environment may provide an incentive to reuse effluent from wastewater treatment plants; however, the city has yet to achieve high proportions of direct recycling, although water coming out of WWTPs is subsequently utilized downstream. Historically there has been high reuse of excreta (i.e., “night soil”) in China (Ashley et al. 2011), but it is unclear if such acceptability remains, especially with the strong drive to modernize and to be a “sanitary” city (Beijing Municipal Government 2014; Jin et al. 2014; Zhang et al. 2015). The fact that Beijing has many of the socio-environmental context factors that have facilitated recycling in other locations, but does not currently exhibit high recycling, supports the idea that no one factor ensures high recycling of urban P.

Our analysis suggests that some “necessary but not sufficient” socio-environmental conditions may underlie P recycling across all cities, but that the unique combination of factors is important to consider for each city. While some socio-environmental factors, such as infrastructure, have clear effects on the practicality and capacity to support P recycling, others are more difficult to evaluate due to limited data and potentially complex relationships. For example, negative perceptions about nutrient recycling from human excreta are an issue for all cities, but the implications of these are not consistent across all case studies (Fig. 3). Even in places where some level of negative perception exists, P recycling still occurs because of economic necessity and water scarcity (e.g., farmers in Accra), or regulations that limit discharge of effluent to water bodies (e.g., London and Baltimore). Additionally, some of the government regulations in these cities are actively addressing public concerns such as odor, pathogens, and heavy metal concentrations in recycled soil amendments. In Buenos Aires on the other hand, negative perceptions about the use of human excreta seem to be strong and have not been overcome, which could be related to biophysical context that allows relatively easy disposal of waste. Further study is needed to draw definitive conclusions about perceptions, but it appears that the effects of negative perceptions may be outweighed by other factors. A central take-home message of our analysis is that interventions to increase P recycling through the urban sanitation chain must account for the local socio-environmental context and address local barriers to P recycling.

Increasing recycling in any city will need to consider how some of the identified key socio-environmental factors may evolve over time, especially with regard to urban sprawl and climate change (Grimm et al. 2008). The distance between urban land uses with high population density (and thus high excreta production) and agricultural lands where it can be applied can act as a major barrier to recycling. In London, the proximity of such land uses facilitated recycling, but sustaining high recycling may be difficult if agricultural land is converted to residential use during urban expansions. In order to keep such proximity, peri-urban agricultural lands would need to be protected. In addition to producing food for urban residents from their own waste, these peri-urban agricultural lands can offer multiple other ecosystem services to urban residents (Zasada 2011).

Even with peri-urban agricultural preservation, transport costs and logistics will need to be considered as cities grow and rural lands change. Human excrement has a high water content and is heavy. Moving it across long distances can be costly, creating a barrier to reuse (as is the case for manure recycling, e.g., Kleinman et al. 2012). Technologies which not only make sure pathogens and other contaminates are removed but also dewater and concentrate nutrients will be necessary to reconnect urban and rural land uses as they move further from each other (Peters and Rowley 2009; Wang et al. 2008).

Lastly, climate change will affect sanitation infrastructure. The siting of new infrastructure and retrofitting of old systems will have to account for sea level rise and flooding. Water availability (e.g., drought) will also impact how much water can be used in sanitation processes (Howard et al. 2010). Climatic changes may affect our ability to recycle P if the risks of P losses from agricultural fields increase. Recommended fertilizer application rates would change and affect how much recycled P can be applied to fields (Ockenden et al. 2016).

### Path dependencies and new opportunities to sustainable P use

Achieving a sustainable urban and agricultural P cycle requires an understanding of which factors are most relevant in a specific context. Acknowledging the historical context of P flow management in a specific city through its sanitation and wastewater practices, norms, and infrastructure can help identify whether strategies for, or barriers to, recycling are transferrable to new situations. For example, in cities such as Baltimore and London, a legacy of combined sewers and aging infrastructure has resulted in untreated sewage being discharged with stormwater. P recovery from waste streams, including sewage overflow, becomes possible when there is political support for investment in infrastructure or technological improvements to direct waste to existing or new treatment facilities, targeting *Junction 5* (sewer to wastewater treatment plans) to increase flows to paths 6f (liquid waste to agricultural fields) and 7f (solid waste to agricultural fields) in Fig. 1. In addition to affecting P recovery, legacies of sewers that combine different waste sources before treatment also influence the problems that might arise when recycling. For example, concerns about microplastic contamination in reused biosolids are a regulatory issue in North America and Europe (Nizzetto et al. 2016).

Importantly, although Baltimore and London currently recycle more P from human excreta than the other case studies considered here, this does not necessarily mean that other cities can or should follow the same development trajectory. Childers et al. (2014) argue that moving beyond centralized sewage and “end of pipe” solutions to incorporate rapidly shifting global environmental, social, and economic contexts experienced by urban ecosystems should and could transform waste/resource management systems towards more sustainable outcomes. Unlike the context of the nineteenth and twentieth century urbanization that shaped London and Baltimore, contemporary climate change as well as other global and local change drivers must be accounted for in the way we govern and plan cities and sanitation (Bulkeley 2010; Rosenzweig et al. 2011; Vörösmarty et al. 2000).

Where sewer infrastructure is minimal or non-existent, such as in Accra or other less industrialized cities, proposals for new construction may take a variety of forms, perhaps focusing on upstream junctions in Fig. 1, because there is no need to address existing aging or failing infrastructure. New planning efforts could design and locate treatment plants to facilitate energy recovery as well as nutrient recycling from biosolids. The planning could consider factors such as proximity to agricultural land, location and magnitude of expected population growth, and the likelihood for inundation under current and future climate and land use scenarios (Harrison et al. 2012). Technological and policy innovations will also continue to evolve, which could further influence appropriate and realistic approaches to P management and recovery in the future. For example, small-scale and decentralized approaches to wastewater treatment are increasingly being identified as a cost-efficient alternative to large, centralized infrastructure in both high- and low-income countries (Cordell et al. 2011; Oakley et al. 2010). These approaches may provide alternative pathways for recycling (such as focusing on pathways to 2f or 3f in Fig. 1) without requiring capital investment in extensive wastewater infrastructure, while still maintaining sufficient protection for human health concerns. As technology development and understanding of the system impacts of different forms of decentralized infrastructure continues to grow, opportunities for P recovery and recycling could develop in unanticipated ways.

In order to increase P recycling rates, local market contexts need to be considered. While centralized infrastructure allows for easier collection of P to apply to agricultural land, it is expensive to build and maintain. Centralized infrastructure is often provided by the public sector, so availability of government funding for such infrastructure projects may partially explain the differences in recycling (Fig. 3). It will be important to identify financing strategies that can cover initial capital costs as well as operation and maintenance costs over the life of the system to provide the multiple functions that sanitation infrastructure should provide. In fact, several studies point towards the potential pitfalls of emphasizing capital investment and new infrastructure at the expense of the continued maintenance, monitoring, and evaluation that is necessary to sustain wastewater treatment services (Mason et al. 2013). In many countries, limited public funds have led to long-term contractual agreements with private companies for the financing, construction, and/or operation of wastewater treatment infrastructure. The effectiveness of privatizing wastewater management activities is mixed, and greater understanding of the long-term consequences of these arrangements is needed (Bloomfield 2006; Hodge and Greve 2007). The degree to which the private sector has been involved in wastewater management differs across countries. For example, public-private partnerships have become mainstream in China (Zhong et al. 2008), while in Buenos Aires, the city’s wastewater management duties have switched from public to private and back to public over time due to a lack of fulfillment of contractual obligations, repeated renegotiations, and fee increases (Ordoqui Urcelay 2007). In some cases, the recovery of nutrients from wastewater can yield revenues sufficient to offset part of the cost of implementing a wastewater management system (De-Bashan and Bashan 2004; Ishii and Boyer 2015; Woods et al. 1999), but may be insufficient to fund the entire operation (Drechsel et al. 2010). Concerted planning, implementation, and capacity building efforts may be required for operational sustainability of these decentralized systems (Molinos-Senante et al. 2010; Parkinson and Tayler 2003).

### Conclusions and next steps for understanding urban P dynamics

Urban P recycling is likely to be a critical component of sustainable development, especially as the world continues to urbanize. However, it is unlikely that a single kind of intervention can maximize urban P recycling across all cities. Our comparison of human excreta P flows through sanitation chains across five globally diverse case study cities illustrates important opportunities and considerations for P recycling. Our research has identified necessary but not sufficient conditions for increased P recycling of human excreta from urban to agricultural areas. In other words, because there are no panaceas, interventions to enhance P recycling through the urban sanitation chain must consider local socio-environmental context. Scientists and policy-makers should consider rapidly changing social and ecological contexts, as well as the legacies of past choices, in order to identify leverage points for sustainable P management. Importantly, P considerations alone are unlikely to provide sufficient motivation for altering urban sanitation systems. The nexus of wastewater, nutrient cycles, energy, greenhouse gas emissions, and human health must all be taken into account to provide holistic solutions. Our approach highlights the path dependencies of large sanitation infrastructure investments in the Global North and contrasts these with rapidly urbanizing cities in the Global South, which present opportunities for new sanitation development pathways for enhanced recycling of P and other nutrients.

## Electronic supplementary material

ESM 1 (DOCX 771 kb)

ESM 2 (DOCX 92.8 kb)
