# Supplementary material for: Socio-environmental consideration of phosphorus flows in the urban sanitation chain of contrasting cities
Source: Reg Environ Change. 2017 Dec 19;18(5):1387–401. doi: 10.1007/s10113-017-1257-7 (PMC6448357; doi:10.1007/s10113-017-1257-7)
Supplement: Supplementary file 1 — (DOCX 771 kb) [file 10113_2017_1257_MOESM1_ESM.docx]

**Supplemental Information**

**Accra, Ghana**

The vast majority of households in Accra have access to improved sanitation (94%, UN Habitat (2016)), where existing infrastructure consists largely of private pit latrines and public latrines. When fecal sludge is collected there is limited treatment before it is disposed of in waterways or on land (Montangero and Strauss 2004, Nikiema et al. 2013, Steiner et al. 2002). A smaller proportion of the population live in houses that are connected to the public sewer system (estimates range from 5-21%), which conveys the sewage to one of several wastewater treatment plants (WWTPs), not likely to be operating as designed (for estimates, see ADF (2015), Boadi and Kuitunen (2005), GSS (2013), UN Habitat (2016)), and potentially treating only 7% of the sewered waste flow (Diener et al. 2014, Van Rooijen et al. 2010). Reports suggest that the remaining waste ends up in the ocean, either directly via dumping, or via conveyance through local gutters and waterways (Burke 2011, Davis 2016).

**Buenos Aires, Argentina**

Access to running water is high (96%), and most households in Buenos Aires have access to water toilets (AySA 2010, PAHO 2001, World Bank 2016). Only about half of the households are, however, connected to the central sewer system, and the remaining half relies on on-site solutions such as pit-latrines, septic tanks or direct discharge to ditches and local rivers. The septic tanks and pit latrines are supposed to be emptied on a regular basis, but it is not uncommon that they are left to overflow, thus accumulating in non-agricultural soils and eventually discharging to local ditches and rivers. When the sludge is pumped out, it is transported to one of the four collection stations, where it is usually discharged untreated (Öberg et al. 2014). Approximately 90% of the sewage that is collected through the central system is discharged untreated into local rivers or Rio de La Plata, which is a large tidal river/ocean bay that discharges into the ocean (AySA 2010, Merlinsky 2013, Rehner et al. 2010). Only a small portion (5-10%) of the sewage that is collected is treated. The solid fraction, including the P it contains, remaining after treatment is trucked and discharged to the local rivers or Rio de la Plata.

**Beijing, China**

The sewer system in the central city is well developed with 96% of the population being connected and conveyed to central WWTPs in 2010 (Qiao et al. 2011, Zhang 2015). The effluent is discharged to the Haihe river system (Pernet-Coudrier et al. 2012, Qiao et al. 2011), which in resent years (e.g., 2006 to 21012, Tang et al. 2015) has had one of the highest P loads of the eight major rivers draining into China's coastal waters. The sludge that is collected is digested anaerobically and then sent to landfill (Qiao et al. 2011, Yang et al. 2015). Qiao et al. (2011) estimated that rural P excreta was either used as fertilizer or disposed of in waterways (and this is reflected in Fig. 3 because, at the time of writing, Qiao et al. (2011) was the most comprehensive quantitative dataset on Beijing P flows). However, others report between 24-75% treatment through a combination of decentralized WWTPs, built wetlands, land infiltration, and unpowered anaerobic treatment processes in these regions (Zhang et al. 2015, Zhang 2015).

The legal status of settlements at the rural fringe of the city is uncertain, and the government could assert authority to develop them at any time. As such, rural residents remain uncertain about potential future compensation rates for such land acquisitions, and in response, they invest minimally in sanitation and other infrastructure and seek fast financial returns in this quasi-legal rental market (Liu et al. 2012). It is not uncommon in these informal settlements for ten apartment units to share a single toilet (Liu et al. 2012).

Baltimore, USA

Most of the sewage is conveyed to two central WWTPs. The system consists of combined sewers that convey both domestic sewage and stormwater runoff. During high flows after large precipitation events, an unknown amount of sewage can escape to the Patapscao Bay waterways and ultimately the greater Chesapeake Bay system in combined sewage overflows (CSOs) (Pelton et al. 2015). The treated WWTP effluent is discharged into the Patapsco and Back Rivers, and the treated sewage sludge is handled as follows: nearly a quarter of the sludge is used as fertilizer on agricultural fields locally or elsewhere in the state of Maryland; another quarter goes to landfill, commercial soil pellet supplements, incineration, or land reclamation; the remainder is exported out-of-state for other diverse uses, such as cement production (Alhija 2008, Maryland Dept. of the Environment 2015).

London, UK

The central sewer system has 100% coverage, and essentially all human excreta P from 8.6 million people in Greater London is conveyed to one of the 350 WWTPs managed by one company, Thames Water (Thames Water Utilities 2016b). The majority of P sequestered in the solid fraction processed at WWTPs is subsequently returned to agricultural soils around the city (approx. 60%-70%, Thames Water Utilities 2016a). The sewage system utilizes CSOs, and, like Baltimore, some P is discharged directly into the surrounding rivers. Although recycling of P has been advancing in London, losses of untreated effluent during peak flows remain a major concern, especially in the face of changing climate and precipitation patterns. Overflow events have been linked to low oxygen and decreased wildlife in the Thames River, and this has motivated construction of the massive Lee Tunnel infrastructure project, which will capture the overflow and route it to treatment facilities (BBC News online 2015) that are expected to be completed by 2020.

Following are tables laying out the data sources for each case study city for the partitioning of P in human excreta and for the socio-environmental factors affecting those P flows. The notes in italics and brackets [] highlight which of the 8 socio-environmental category/domain affecting P (as defined by Metson et al. 2015) each factor primarily belongs to.

**ACCRA, GHANA**

| **Junction** | **Partition value or flow quantity** | **References** | **SE factors affecting P flows** | **References** |
| --- | --- | --- | --- | --- |
| 1 | 3-6% of population has no access to sanitary facilities  15-37% of population (depending on reference) has access to a flush toilet.  Additional 60% of population have access to some form of latrine (public or private). | Boadi and Kuitunen (2005)  Benneh 1993, Boadi and Kuitunen (2005), ADF (2005),  UN Habitat (2016)  Boadi and Kuitunen (2005) | The high cost of sanitation infrastructure, and the presence of more pressing issues (shelter, water, food, and school) are barriers to expanding and improving sanitation in Ghana [*Future priorities and plans*]  Access to toilets and disposal of waste highly stratified by wealth [*Market and capital availability*] | Nimoh et al. (2014), Card and Sparkman (2010)  Boadi and Kuitnunen (2005) |
| 1c (no toilet to random soil) | 100% of population with no toilet (3-6% of population) disposes of waste in yards or roads | Boadi and Kuitunen (2005) |  |  |
| 2 | 60% of population uses latrines (uncontained), either public or private | Boadi and Kuitunen (2005) |  |  |
| 2b | 28% of waste (47% of latrine waste) is disposed of in freshwater systems or gutters | Boadi and Kuitunen (2005) |  |  |
| 2c | 32% of waste (53% of latrine waste) is disposed of in unproductive soils (yards, roads) | Boadi and Kuitunen (2005) |  |  |
| 4 – septic vs sewered | 5-21% of population served by sewers (24-100% of toilet waste)  8.6% of population served by septic  Some septic systems are pumped and waste carried to WWTPs | Cofie (2009), ADF (2005), GSS (2013)  Boadi and Kuitunen (2005)  Montanegro and Strauss (2004) |  |  |
| 5 - untreated sewer vs WWTP | 5-7% of sewered wastewater is treated (WWTP capacity is only 7% of need) | Van Rooijen et al. (2010), Akuffo (2001) | Ongoing infrastructure investment in sewers and treatment facilities means these numbers are changing quickly [*Infrastructure and landuse*] | ADF (2005) |
| 5,6a | 95-100% of wastewater dumped in the lagoon (ultimately ocean) | Akuffo (2001), Van Rooijen (2010) | The Korle lagoon has been subject to NGO (including the UK) plans for environmental cleanup and a new WWTP, but without taking into consideration unplanned settlements these efforts don’t seem to be making a difference yet [*Biogeophysical situation, Governance and actors*] | Hofny-Collins (2006) |
| 7 | faecal sludge treatment does exist, but capacity, treatment levels, and fate unknown |  | Challenges reported for WW treatment include:  - technical challenges (power failure, power cuts, overloading) [*Infrastructure and landuse*]  - social challenges (waste thrown in sludge  complaints about odor and mosquitos) [ *Cultural norms and preferences*]  - economic challenges (lack of funds and high costs for operations and maintenance) [*Market and capital availability*]  - environmental challenges (odor affecting locals in vicinity) [*Infrastructure and landuse*]  Variety of technical challenges with faecal sludge treatment plants, including high ammonium concentrations and unsafe levels of parasites in treated sludge. Three faecal sludge treatments plants are no longer functioning as of 2014. [*Infrastructure and landuse*] | Nikiema et al. (2013)  Steiner (2002), Diener et al. (2014) |
| 7a | 100% of faecal sludge disposed of at beach (at single official faecal sludge dumping site) and ends up in ocean | Diener et al. (2014) | Beach disposal started after the collapse of an outfall. The situation has been improved after completion of the Accra Waste Project and the Korle Lagoon Environmental Restoration Project (KLERP) [*Infrastructure and landuse*] |  |
| 1,2,3,4,5f | ~7% of waste ends up being recycled from freshwater systems (where it is disposed of, untreated) to urban agricultural fields | Van Rooijen et al. (2010) | Water scarcity is driving some reuse of water (and therefore waste) [*Biophysical situation*]  High costs of fertilizers for farmers may also be driving reuse [*Market and capital availability*]  Farmers in peri-urban Accra (Shai-Osudoku) view human excreta as a resource for agriculture but also realize that it can be a health risk and this can thus limit reuse [*Knowledge and access to information*]  Reuse is restricted by laws and there are (accurate) perceptions that it presents a health risk [*Government and regulations*]  Younger farmers who own their land are more likely to be willing to reuse fecal sludge [*Governance and actors*]  In order to increase safe recycling there needs to be a profitable business model and so far that has not been the case (does not seem feasible without a government subsidy) [*Market and capital availability*] | Owusu et al. (2012), Cofie et al. (2010)  Nimoh et al. (2014)  Danso et al. (2006), Drechsel et al. (2010) |

**BUENOS ARIES, ARGENTINA**

| **Junction** | **Partition value or flow quantity** | **References** | **SE factors affecting P flows** | **References** |
| --- | --- | --- | --- | --- |
| 1 | 96% of the urban population in Argentina had access to improved sanitation facilities in 2015 (includes flush/pour flush, ventilated improved pit latrine, pit latrine with slab, and composting toilet) | World Bank (2016) |  |  |
| 5 | 47% of the population of the Buenos Aires Metropolitan area had access to sewers in 2010 | Nabel (2010) | Lack of progress in expanding sewer connectivity due to institutional fragmentation and instability [*Government & Regulation, Governance & Actors*] | Ordoqui Urcelay (2007), Botton and de Gouvello (2008), Merlinsky (2013), Oberg et al. (2014), Morales et al. (2014) |
| 6, 7 | 5.3 to 10% of effluent was treated from sanitary sewers in 2001 | PAHO (2001), Rehner, Samaniego, and Jordán Fuchs (2010) | Proximity to local rivers with high flow rates that discharge into the ocean allow for minimal wastewater treatment [*Biogeophysical*]  Lack of progress in expanding wastewater treatment due to institutional fragmentation and instability [*Government & Regulation, Governance & Actors*] | Ordoqui Urcelay (2007), Botton and de Gouvello (2008), Merlinsky (2013), Oberg et al. (2014), Morales et al. (2014) |
| 4 | 31% of the population of the Buenos Aires Metropolitan area has access to in situ sanitation with septic tank in 2010 | Nabel (2010) |  |  |
|  |  |  | Land application of recycled human excreta is not a socially acceptable practice [*Cultural Norms & Preferences*] | Merlinsky, pers comm. |

**BEIJING, CHINA**

| **Junction** | **Partition value or flow quantity** | **References** | **SE factors affecting P flows** | **References** |
| --- | --- | --- | --- | --- |
| 1b,f  2b,f | Population classified as 84.9% urban and 15.1% rural (i.e., rural suburbs) by Qiao et al. (2011)  Percent toilets is inferred at 96% toilet use in urban areas and lower use rate in rural areas. Fig. 3 in the main text is based off of Qiao et al. (2011), who models all rural excreta directly to environmental sinks. However, see discussion in text of alternative waste management in suburbs.  Qiao et al. (2011) model 11.1% urban flow (9% of total flow) going directly to the fresh water system and 10% (8% of total flow) going directly to agriculture. | Qiao et al. (2011), Zhang (2015), Zhang et al. (2015), Beijing Municipal Government (2014), Hu (2015) | The Beijing government has a strong development mandate to be a clean and modern city. [*Future priorities and plans*]  While pushing to achieve a high level of sewage hookup in the city center, there is uneven development spatially and infrastructure is lacking in areas occupied by a marginalized present class (see discussion in text) [*Governance and actors*]  China as a whole has a long history of using excreta directly as fertilizer, though this is changing due to concerns about pharmaceutical, heavy metal, and other contaminants [*Cultural norms and preferences*]  Public toilets could be part of a sanitation solution where comprehensive household sewage hookups may be unfeasible. Beijing has a long history of providing and using public toilets for collecting human excreta and recent news reports suggest that this trend will continue. [*Future priorities and plans*] | Liu et al. (2012),  Beijing Municipal Government (2014)  Liu et al. (2012)  Murray et al. (2011), Qiao et al. (2011), Chen et al., (2012) Jin et al. (2014)  (Geisler 2000), (Xinhua News 2015) |
| 6b,d | 78.9% of urban flow (68% of total flow) goes to WWTP. 10.3% of this urban P flow (7% of total) is discharged to the river system, 14% of it (9% of total) is applied to urban green spaces. | Qiao et al. (2011) | The Beijing government has a strong development mandate to be a clean and modern city. Hosting 2008 Olympic games was a catalyst to develop modern WWT infrastructure. [*Future Priorities and plans*]  Beijing government regulates that effluent contains a maximum of 0.6 mgP/L [*Government and regulation*]  Biophysical setting of drought has also led to water reclamation policies, which affect wastewater infrastructure and reuse to other applications [*Biogeophysical situation, Future priorities and plans*] | Beijing Municipal Government (2014)  Kuang (2012),  Pernet-Coudrier et al. (2012)  Tong et al., (2015)  Sun et al. (2014), Zhang et al. (2015), Jin et al. (2014) |
| 7e | 75.7% of urban P flow (51% of total) goes to landfill. | Qiao et al. (2011) | The vast majority of P entering the wastewater system ends up in landfill.  China has a large domestic supplies of phosphorus rock, which likely disincentivizes P recycling (e.g., diversion away from landfill) [*Biogeophysical situation, Market and capital availability*]  Other factors that complicate recycling of solid fraction include the fact that sewage is contaminated with pharmaceuticals, personal care products, industry surfactants, and heavy metals. P recycling is further complicated by an inability to source separate industrial and domestic waste streams. [*Infrastructure and landuse, Government and regulations, Cultural norms and preferences*]  On the other hand, Industrial applications, such as cement manufacturing, which China has a huge appetite for, could encourage recycling [*Market and capital availability*] | Qiao et al. (2011),  Yang et al., (2015)  Zhou et al. (2016)  Jin et al. (2014), Li et al. (2016)  Chen et al. (2012) |

**BALTIMORE, USA**

| **Junction** | **Partition value or flow quantity** | **References** | **SE factors affecting P flows ([*8 dimensions* ])** | **References** |
| --- | --- | --- | --- | --- |
| 1,2,3,4 | 0% | Baltimore County Dept. of Planning (2015) and Baltimore City (2012). | In greater Baltimore County, there are about 93,000 residences that are on private septic systems. The installation of sewers and private septic systems is regulated by the Maryland Department of Planning Sustainable Growth and Agricultural Preservation Act of 2012 (commonly known as the Septics Law) which designates areas into one of four categories (tiers) to limit installation of septic systems on large lot residential developments. More recently, amendments to this law discourage septic systems in suburban/rural areas surrounding Baltimore City by setting and enforcing environmental targets, not mandating particular technologies for meeting these goals. Taking this zoning approach also restricts development of major subdivisions in agricultural and forest areas and concentrate future growth in areas serviced by sewer [*Government and regulation, Infrastructure*] | General Assembly of Maryland (2012). Wainger (2016). |
| 5a, 5b | 22-330 million gallons lost per year | Environmental Integrity Project (2015) and Maryland Dept. of the Environment (2016) | The City of Baltimore is now under a legal obligation to the state of Maryland and the federal government to evaluate and rehabilitation its wastewater collection system based on a Consent Decree. Although this has resulted in much repair of aging infrastructure, funding is still inadequate and hence overflows remain a problem (legacy of combined sewage system). [*Infrastructure and Land Use, Government and Regulation*] | United States District Court (2002). |
| 6 | 0%. Monthly average of WWTP effluent must be below 0.2 mg/l. | Chow (2014), EPA (2014). | All wastewater collected in the sewer system flows to one of two wastewater treatment plants: Back River or Patapsco. Back River treats approximately 180 million gallons per day, and Patapsco treats approximately 63 million gallons per day. These plants have a high efficiency of removing P from liquid outflow. [*Infrastructure, Knowledge and access to information, Cultural Norms and preferences, Government and Regulation, Market and Capital Availability*] | United States District Court (2002). |
| 7 | Nearly 100% (> 700,000 WT of sludge) | Maryland Dept. of the Environment (2015b), Chow (2014). | Maryland Planning Department Septics Law assures all sewage sludge is collected at these two plants, see description below [*Government and regulation, Infrastructure*] | General Assembly of Maryland (2012). |
| 7c | 1-4% annually (from 2010 - 2014), including marginal usage | Maryland Dept. of the Environment (2015a, 2015b) | Since 1974, the Maryland Department of the Environment issues permits for the utilization of sewage sludge, which is also regulated at a federal level by the Clean Water Act Section 503. Permit approval is contingent on demonstration that the utilization will not cause risk to the environment or public health, welfare, or safety which requires permitees to provide detailed information on their plans for utilization and the site of application including a nutrient management plan. The permit approval process also includes a public hearing in which concerns can be raised. At these public hearings, public opposition has included issues of smell, property values, environmental impacts, and noise, however applications are rarely denied solely on the basis of public opposition. (However several permits have been withdrawn or denied from public opposition.) MDE has issued over 5,000 sewage sludge permits since 1974. [*Knowledge and access to information, Cultural Norms and preferences, Government and Regulation, Market and Capital Availability*] | COMAR Regulation 26.04.06. US EPA (2016). |
| 7d | 10-13% annually (from 2010 - 2014), includes distributed and marketed as well as incinerated. | Maryland Dept. of the Environment (2015a, 2015b), Alhija (2008) | Other productive uses include commercial soil pellets, land reclamation, and combustion for energy [*Knowledge and access to information, Cultural Norms and preferences, Government and Regulation, Market and Capital Availability*] | COMAR Regulation 26.04.06. US EPA (2016). |
| 7e | 9-13% annually (from 2010 – 2014), includes landfill utilization/disposal and storage. | Maryland Dept. of the Environment (2015a, 2015b), Alhija (2008) | COMAR zoning regulations restrict where biosolids can be stored and US EPA regulates runoff from these sites. [*Knowledge and access to information, Cultural Norms and preferences, Government and Regulation, Market and Capital Availability*] | COMAR Regulation 26.04.06. US EPA (2016). |
| 7f | 8-24% annually (from 2010 - 2014) | Maryland Dept. of the Environment (2015a, 2015b), Alhija (2008) | About 43,000 acres on about 300 farms throughout Maryland are currently permitted to receive sewage sludge. Transportation of sewage sludge raises an issue because COMAR permits to transport sludge require transporting sludge directly from treatment facilities to farms, therefore farmers need to be ready to apply sludge when it arrives. In addition to these state restrictions, the EPA also regulates the utilization of biosolids on ag fields through the Clean Water Act [*Knowledge and access to information, Cultural Norms and preferences, Government and Regulation, Market and Capital Availability*] | COMAR Regulation 26.04.06. US EPA (2016). |
| 7x | 49-68% annually to unknown sink (from 2010 - 2014), includes out-of-state and other | Maryland Dept. of the Environment (2015a, 2015b), Alhija (2008) | The permit approval process for sewage sludge utilization includes a public hearing where concerns can be raised. At public hearings, public opposition has included issues of smell, property values, environmental impacts, and noise, resulting in several permits having been withdrawn or denied. A major use of sludge transported out of state is a cement-making plant. [*Knowledge and access to information, Cultural Norms and preferences, Government and Regulation, Market and Capital Availability*] | COMAR Regulation 26.04.06. US EPA (2016), Hare (2007) |

*note on system boundaries: The city of Baltimore has two wastewater treatment plants, but the data regarding the fate of sewage sludge comes at the state level from the Maryland Dept. of the Environment (2015). As such, we assume that Baltimore waste is proportionally used the same was as the whole state of Maryland.

**LONDON, ENGLAND**

| **Junction** | **Partition value or flow quantity** | **References** | **SE factors affecting P flows ([*8 dimensions* ])** | **References** |
| --- | --- | --- | --- | --- |
| 1,2,4,5 | 100% of population using toilets and connected to centralized sewage | The World Bank (2016)  DEFRA (2012)  Global City Institute (2016) | Historically concerned with city livability and human health (cholera outbreaks and smell) [*Cultural norms and preferences*]  London is an early adopted in implementing such extensive sewage collection and treatment after the historical events [*Infrastructure, Access to capital and markets*] | Cicak and Tynan (2015) |
| 5b | -Aprox. 10% loss based on tertiary treatment of sewage  -Aprox. 1% loss to sewage overflow (Thames water treats 1,022,000,000 cubic meters and 12 million are lost to overflow) | Powers et al. (2016) SI materials, Thames Water Utilities (2016)  The Guardian (2005) and BBC (2015) | History of combined sewage (stormwater runoff and wastewater are combined) [I*nfrastructure and land use*]  Proximity of a larger river makes this type of waste management possible [*Biogeophysical situation*]  All WW management is done through one company Thames Water Utilities [*Governance and actors*] | The Guardian (2005), BBC (2015) |
| 6b |  |  | All WWTP in London have at least tertiary treatment so little P is making it to waterways. [*Infrastructure and land use*]  There is a vast suite of national and EU laws that direct how human excreta is to be disposed of and how biosolids and other recycled products can be reused. This includes the Sewage Sludge Directive 86/278/EEC and the UK level, as well as the Nitrate Directive and Urban Wastewater Treatment Directive 91/271/EEC at the EU level, and the Sludge (Use in Agriculture) Regulations 1989 [*Government and Regulation*]  Some of these regulations are to protect the environment while others are to protect human health, as well as take into account the perception of citizens around sewage treatment and reuse [*Knowledge and access to information, Cultural Norms and preferences*] | Thames Water Utilities (2016)  Thames Water Utilities (2008), EU (2016) |
| 7f | 60-70% with aprox. 1 % processed as a fertilizer product | Thames Water Utilities (2008)  (note that the company reports different %, up to 90% TWU (2016c)  BBC (2013) | Proximity to agricultural land helps recycling [*Infrastructure and land use*]  Application rates are regulated by national and EU laws and industry standards including the Safe Sludge Matrix [*Government Laws and Regulation*]  Public perception is an issue considered in regulation and how Thames Water and farmers manage biosolids may become an issue [*Knowledge and access to information, Cultural Norms and preferences*]  Has 1 WWTP that has a partnership with Ostara that produces struvite crystals [*Knowledge and access to information*] | Thames Water Utilities (2008), Kelly et al. (2002)  Thames water Utilities (2016d)  BBC (2013) |
| 7e | 0 |  | It is illegal to landfill biosolid products [*Government and Regulation*] | EU (1999) |
| 7d | 20% |  | -Energy production from WWTP has been a priority for Thames Water (6 of the WWTP have thermal hydrolysis) but the ash from the incineration process is harder to reuse meaning there is potentially some landfilling or at least reuse in land reclamation instead of to agriculture. [Future priorities and plans, technology]  -The technology of incineration when there are other metals and substances that stay in the ash made the recycling to agriculture difficult [technology] | Thames Water Utilities (2016c)  Thames Water Utilities (2008), Thames Water Utilities (2016b) |

*note on system boundaries: Thames water have 350 treatment plants and covers more than the Greater London region proper (the company treats waste for 15 million people and Greater London is a little over 8 million people) but we did not have enough easily available site specific information to determine which plants covered London vs the surrounding area and how that may change the fate of waste flows. As such we assume that London waste is proportionally treated the same was as the whole Thames Water Utility network. The Cambi plant (serving 3.5 million people, which does serve the London area and recycles treated biosolids to nearby agricultural lands (WaterWorld (2013)).

**Box SI1**. Definitions used in the sanitation chain.

*Sewage*: Present-day usage commonly refers to waterborne waste, normally conveying human excreta. Depending on the layout of the sewer system, it may also include industrial wastewater and precipitation.

*Sewer*: Infrastructure that conveys sewage. It is worth noting that the introduction of sewers in the English-speaking world predates the introduction of water toilets, and that, originally, sewers consequently did not convey blackwater (toilet water).

*Combined sewers (CSs)*: Sewers that convey both domestic sewage (blackwater and greywater) as well as precipitation. During peak-flows (caused by for example rain-storms) the sewers are unable to remove water fast enough. To avoid overflowing into city streets, the system has ‘overflows’ that discharged directly into waterways without first passing treatment plants. This means that heavily diluted but untreated sewage can be discharged directly to waterbodies at times through these combined sewer overflow (CSO) events.

*Sewage treatment*: A summary term used to denote various (technical) approaches to reduce problems related to waterborne waste, such as odor, pathogens, nutrients, heavy metals, persistent organic pollutants (POPs), pharmaceuticals, and personal care products (PCPs).

*Sewage sludge*: When sewage is treated in a treatment plant, it results in two products: effluent = wastewater and a semisolid residual = sewage sludge.

*Biosolids/treated sewage sludge*: If further treated, sewage sludge is called “biosolids" in North American regulations and "treated sewage sludge" in European regulations.

*Wastewater*: Water that is discharged after some kind of treatment, i.e. effluent from a treatment plant (domestic or industrial). Wastewater is commonly used synonymously with sewage, which can be rather confusing.

*Septic systems:* Storing waterborne human excreta in a pond or a tank results in a relatively clean effluent with respect to pathogens, and it also reduces odor this is not a definition rather the process. In addition to being a common component of conventional sewage treatment plants, it is the basic principle for septic tanks. The effluent from a septic tank is led to a septic field where the water is filtered through a plant-soil system, which further cleans the water.

*Seepage*: The term is used to describe uncontrolled discharge of effluent from pit-latrines and mismanaged septic systems. In such cases, the effluent ‘seeps’ into the environment and, instead of being filtrated through a soil-water system, or collected by a truck, the content is allowed to overflow directly to streets, ditches and local watercourses.

*Fecal sludge*: The slurry of feces and urine that is formed in a pit-latrine. In most cases, these toilets are so called pour-flush which means that the person using the toilet uses a bucket with water to clean themselves after using the toilet. The water content of fecal sludge is thus normally considerably lower than in systems that use water flush toilets. Fecal sludge is at times used to denote the semi-solid material remaining in a septic tank.

*Soil amendment*: Anything that is added to soil with the intent of improving the quality of the soil from agricultural point of view.

**Box SI2**. Using the GLOBE tool to explore city diversity

A promising tool for illustrating and understanding socio-environmental diversity across cities is provided by the GLOBE collaboration engine ([www.ecotope.org)](http://www.ecotope.org)). This tool leverages a large library of globally contiguous, standardized data sets, enabling rapid comparisons among grid cells located anywhere in the world. As an initial step towards understanding differences among the case study cities, we used this standardized data to conduct a GLOBE similarity analysis. In this initial effort, we chose the following P-relevant urban and agricultural variables, using grid cells within a 100 km buffer of each city center:

Percent cropland within the landscape, as an indicator of the capacity to recycle P close to the urban center

Urban and built-up areas within the landscape, as an indicator of the sanitation infrastructure and how P may move in the city

Soil suitability class (for agriculture), as an indicator of the capacity for sustainable agriculture

Population density trend between 1950 & 2000, as an indicator of historical changes in human waste production and urbanization

N fertilizer use, the only fertilizer variable yet available in GLOBE, as a coarse indicator of P fertilizer demand, and also wealth

Not surprisingly, when we compared London (a ‘moderately dense’ city among land parcels worldwide, with >100 people km^-2^) with the other cities, we found that all grid-cells in Buenos Aires, Beijing, and Baltimore were dissimilar to London (lower than 50^th^ percentile for the ‘similarity’ metric). While the Accra region had multiple grid cells that were moderately similar to London (75-90% percentile for similarity), these were adjacent to large swaths of dissimilar land, partly reflecting lower topographic suitability for development (due to higher slope and relief) compared to London. These results reinforce our interests, and motivations for our selected case study approach.

**Figure SI1**. Socio-environmental highlights for the 5 case study cities. A. Global positioning and population density (GRUMP-SEDAC 2009) B. Accra, Ghana C. Buenos Aires, Argentina D. Beijing, China E. Baltimore, USA F. London, England. Land use data are from the European Space Agency (Arino et al. 2012). Hydrologic network and topography layers^[[1]](#footnote-1)^ were made with Natural Earth (free vector and raster map data @ naturalearthdata.com). Population data are from multiple sources (Accra: Buenos Aires: (Öberg et al. 2014), Accra: (Government of Ghana 2016, GSS 2013), Beijing: (National Bureau of Statistics of China 2011), Baltimore: (U.S Census Bureau 2016), London: (ONS 2016)), and gross domestic product (GDP) are in USA$(World Bank 2016)^[[2]](#footnote-2)^.


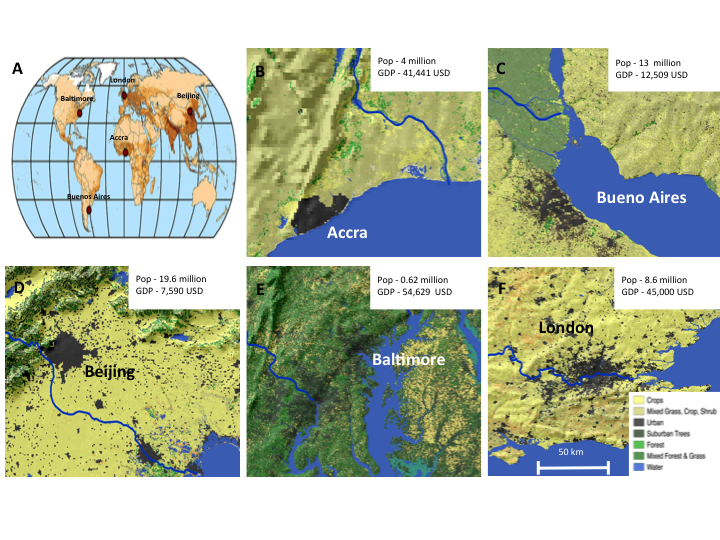


1. We selected a few socio-environmental variables that could affect P flows in cities. We represented land-use mix as it gives an idea of the proximity of agricultural lands that could be available for recycling, as well as urban land use form which could affect sanitation infrastructure. We represent hydrology (presence of water ways in the maps) as it can be a receiving system of P from sanitation systems and agricultural lands. We represent topography as it can affect sanitation infrastructure as well as land use and capacity to transport P back to productive land-uses. [↑](#footnote-ref-1)
2. http://data.worldbank.org/data-catalog/GDP-ranking-table [↑](#footnote-ref-2)
