# Supplementary material for: Socio-environmental consideration of phosphorus flows in the urban sanitation chain of contrasting cities
Source: Reg Environ Change. 2017 Dec 19;18(5):1387–401. doi: 10.1007/s10113-017-1257-7 (PMC6448357; doi:10.1007/s10113-017-1257-7)
Supplement: Supplementary file 2 — (DOCX 92.8 kb) [file 10113_2017_1257_MOESM2_ESM.docx]

**Table 1.** Data sources for each case study city for the partitioning of P in human excreta (Figure 2) and for the socio-environmental factors affecting those P flow. Not every node is applicable to each city; these nodes are indicated by “--”.

| **ACCRA, GHANA** | | |
| --- | --- | --- |
| **Junction** | **References for partition value of P flow quantity** | **References for SE factors** |
| 1, 2 | Boadi and Kuitunen (2005), Benneh 1993, Boadi and Kuitunen (2005), ADF (2005),  UN Habitat (2016) | Nimoh et al. (2014), Card and Sparkman (2010), Boadi and Kuitnunen (2005) |
| 4 | Cofie (2009), ADF (2005), GSS (2013), Boadi and Kuitunen (2005), Montanegro and Strauss (2004) | **--** |
| 5 | Van Rooijen et al. (2010), Akuffo (2001) | ADF (2005) |
| 5,6a | Akuffo (2001), Van Rooijen (2010) | Hofny-Collins (2006) |
| 7 | -- | Nikiema et al. (2013), Steiner (2002), Diener et al. (2014) |
| 7a | Diener et al. (2014) | -- |
| 1,2,3,4,5f | Van Rooijen et al. (2010) | Owusu et al. (2012), Cofie et al. (2010), Nimoh et al. (2014), Danso et al. (2006), Drechsel et al. (2010) |
| **BUENOS ARIES, ARGENTINA** | | |
| 1 | World Bank (2016a) | **--** |
| 5 | Nabel (2010) | Ordoqui Urcelay (2007), Botton and de Gouvello (2008), Merlinsky (2013), Oberg et al. (2014), Morales et al. (2014) |
| 6, 7 | PAHO (2001), Rehner, Samaniego, and Jordán Fuchs (2010) | Ordoqui Urcelay (2007), Botton and de Gouvello (2008), Merlinsky (2013), Oberg et al. (2014), Morales et al. (2014) |
| 4 | Nabel (2010) | -- |
| f | -- | Merlisky pers. Comm. (Pr. sociology University of Buenos Aires) and Merlisky (2013) |
| **BEIJING, CHINA** | | |
| 1b,f  2b,f | Qiao et al. (2011), Zhang (2015), Zhang et al. (2015), Beijing Municipal Government (2014), Hu (2015) | Liu et al. (2012), Beijing Municipal Government (2014), Murray et al. (2011), Qiao et al. (2011), Chen et al., (2012) Jin et al. (2014), (Geisler 2000), (Xinhua News 2015) |
| 6b,d | Qiao et al. (2011) | Beijing Municipal Government (2014), Kuang (2012), Pernet-Coudrier et al. (2012), Tong et al., (2015), Sun et al. (2014), Zhang et al. (2015), Jin et al. (2014) |
| 7e | Qiao et al. (2011) | Qiao et al. (2011), Yang et al., (2015), Zhou et al. (2016), Jin et al. (2014), Li et al. (2016), Chen et al. (2012) |
| **BALTIMORE, USA** | | |
| 1,2,3,4 | Baltimore County Dept. of Planning (2015) and Baltimore City (2012) | General Assembly of Maryland (2012). Wainger (2016) |
| 5a, 5b | Environmental Integrity Project (2015) and Maryland Dept. of the Environment (2016) | United States District Court (2002). |
| 6 | Chow (2014), EPA (2014). | United States District Court (2002). |
| 7 | Maryland Dept. of the Environment (2015b), Chow (2014) | General Assembly of Maryland (2012) |
| 7c, d, e, f, x | Maryland Dept. of the Environment (2015a, 2015b), Alhija (2008) | COMAR Regulation 26.04.06. US EPA (2016), Hare (2007) |
| **LONDON, ENGLAND** | | |
| 1,2,4,5 | The World Bank (2016ab), DEFRA (2012), Global City Institute (2016) | Cicak and Tynan (2015) |
| 5b | Powers et al. (2016) SI materials, Thames Water Utilities (2016), The Guardian (2004), BBC (2015) | The Guardian (2004), BBC (2015) |
| 6b | -- | Thames Water Utilities (2016a), Thames Water Utilities (2008), EU (2016) |
| 7f | Thames Water Utilities (2008), TWU (2016c), BBC (2013) | Thames Water Utilities (2008), Kelly et al. (2002), Thames water Utilities (2016d), BBC (2013) |
| 7e | -- | EU (1999) |
| 7d | -- | Thames Water Utilities (2016c),Thames Water Utilities (2008), Thames Water Utilities (2016b) |
